# Increased Serum Levels of Anti-Carbamylated 78-kDa Glucose-Regulated Protein Antibody in Patients with Rheumatoid Arthritis

**DOI:** 10.3390/ijms17091510

**Published:** 2016-09-08

**Authors:** Hui-Chun Yu, Pei-Hsuan Lai, Ning-Sheng Lai, Hsien-Bin Huang, Malcolm Koo, Ming-Chi Lu

**Affiliations:** 1 Department of Medical Research, Dalin Tzu Chi Hospital, Buddhist Tzu Chi Medical Foundation, Dalin, Chiayi 62247, Taiwan; df928039@tzuchi.com.tw (H.-C.Y.); m.koo@utoronto.ca (M.K.); 2 Division of Allergy, Immunology and Rheumatology, Dalin Tzu Chi Hospital, Buddhist Tzu Chi Medical Foundation, Dalin, Chiayi 62247, Taiwan; df587462@tzuchi.com.tw (P.-H.L.); tzuchilai@gmail.com (N.-S.L.); 3 School of Medicine, Tzu Chi University, Hualien 97004, Taiwan; 4 Department of Life Science and Institute of Molecular Biology, National Chung Cheng University, Minxiong, Chiayi 62102, Taiwan; biohbh@ccu.edu.tw; 5 Dalla Lana School of Public Health, University of Toronto, Toronto, ON M5T 3M7, Canada

**Keywords:** GRP78, autoantibodies, rheumatoid arthritis, systemic autoimmune diseases

## Abstract

The objective of this study was to investigate the presence and titer of anti-carbamylated 78-kDa glucose-regulated protein (anti-CarGRP78) antibody in serum from controls, and patients with rheumatoid arthritis (RA), systemic lupus erythematosus (SLE) and primary Sjögren syndrome (pSS). Thirty-three RA patients, 20 SLE patients, 20 pSS patients, and 20 controls were enrolled from our outpatient clinic. GRP78 was cloned and carbamylated. Serum titers of anti- cyclic citrullinated peptides (anti-CCP), anti-GRP78, and anti-CarGRP78 were measured with an enzyme-linked immunosorbent assay. No differences in serum titers of anti-GRP78 antibody in patients with RA, SLE, or pSS compared with the controls were observed. Serum levels of anti-carGRP78 antibody in patients with RA, but not SLE or pSS, were significantly higher compared with the controls (OD_405_ 0.15 ± 0.08 versus 0.11 ± 0.03, *p* = 0.033). There was a positive correlation between the serum levels of anti-GRP78 antibody, but not anti-CarGRP78 antibody, with the levels of anti-CCP antibody in patients with RA. Both anti-GRP78 and anti-carGRP78 antibodies failed to correlate with C-reactive protein levels in patients with RA. In conclusion, we demonstrated the presence of anti-CarGRP78 antibody in patients with RA. In addition, the serum titer of anti-CarGRP78 antibody was significantly elevated in patients with RA compared with the controls. Anti-CarGRP78 antibody could also be detected in patients with SLE or pSS.

## 1. Introduction

The 78 kDa glucose-regulated protein (GRP78), also known as binding immunoglobulin protein (BiP), has been shown to be an important autoantigen for rheumatoid arthritis (RA). GRP78 could stimulate the proliferation of synovial T cells from patients with RA [1]. Anti-GRP78 antibody can be found in the serum from patients both after and before their diagnosis of RA [2]. Anti-citrullinated protein antibody (ACPA) is known to be a very specific diagnostic marker for RA. In our previous study, we found that GRP78 was citrullinated (citGRP78) and became recognizable by ACPAs, and ACPAs could directly stimulate monocytes to secrete tumor necrosis factor α (TNF-α) via binding to citGRP78 [3]. In addition, anti-citGRP78 antibody was also widely detected in serum from patients with RA [4]. Moreover, parenteral administration of GRP78 could also ameliorate joint inflammation in mice with collagen-induced arthritis [5].

Carbamylation is a post-translational modification in which cyanate binds to primary amino or thiol groups. Recently, autoantibodies recognizing carbamylated proteins (anti-CarP antibodies) were found to be present in the sera from patients with RA and were predictive for joint damage [6]. In addition, the presence of anti-CarP antibodies could predict the development of RA [7] and serve as a poor prognostic factor for long-term disability in patients with RA [8]. Although the initial antigen used for detecting anti-CarP antibodies was carbamylated fetal calf serum [6], other carbamylated antigens such as α-enolase or vimentin [9,10] were found to be part of the anti-CarP antibodies. It is conceivable that other proteins could be carbamylated and recognizable by anti-CarP antibodies. Therefore, we hypothesized that the anti-CarGRP78 antibody might be present in the serum from patients with RA. The serum titers of anti-CarGRP78 antibody among patients with other systemic autoimmune diseases, including systemic lupus erythematosus (SLE) and primary Sjögren syndrome (pSS), were also investigated in this study.

## 2. Results

### 2.1. Patients and Controls

We recorded the demographic and clinical characteristics of controls and patients with RA, SLE or pSS, including age, sex, anti-cyclic citrullinated peptides (anti-CCP) positivity, and rheumatoid factor positivity (Table 1).

### 2.2. Validation of Carbamylaion of GRP78

**Figure 1:**
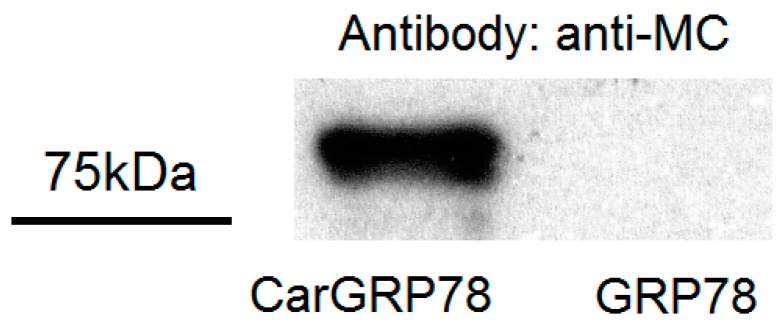
Verification of the carbamylated GRP78 (CarGRP78) by Western blotting. Cloned GRP78 and CarGRP78 were modified by 2,3-butanedione monoxime and antipyrine in a strong acid solution. The modified citrulline residues were detected with rabbit polyclonal anti-modified citrulline antibodies. Only the CarGRP78 reacted with anti-modified citrulline antibodies.

The carbamylated GRP78, but not the native form GRP78, was recognizable by anti-modified citrulline antibody (Figure 1).

### 2.3. Serum Titers of Anti-GRP78 and Anti-CarGRP78 Antibody among Controls and Patients with Rheumatoid Arthritis, Systemic Lupus Erythematosus, or Primary Sjögren’s Syndrome

**Figure 2:**
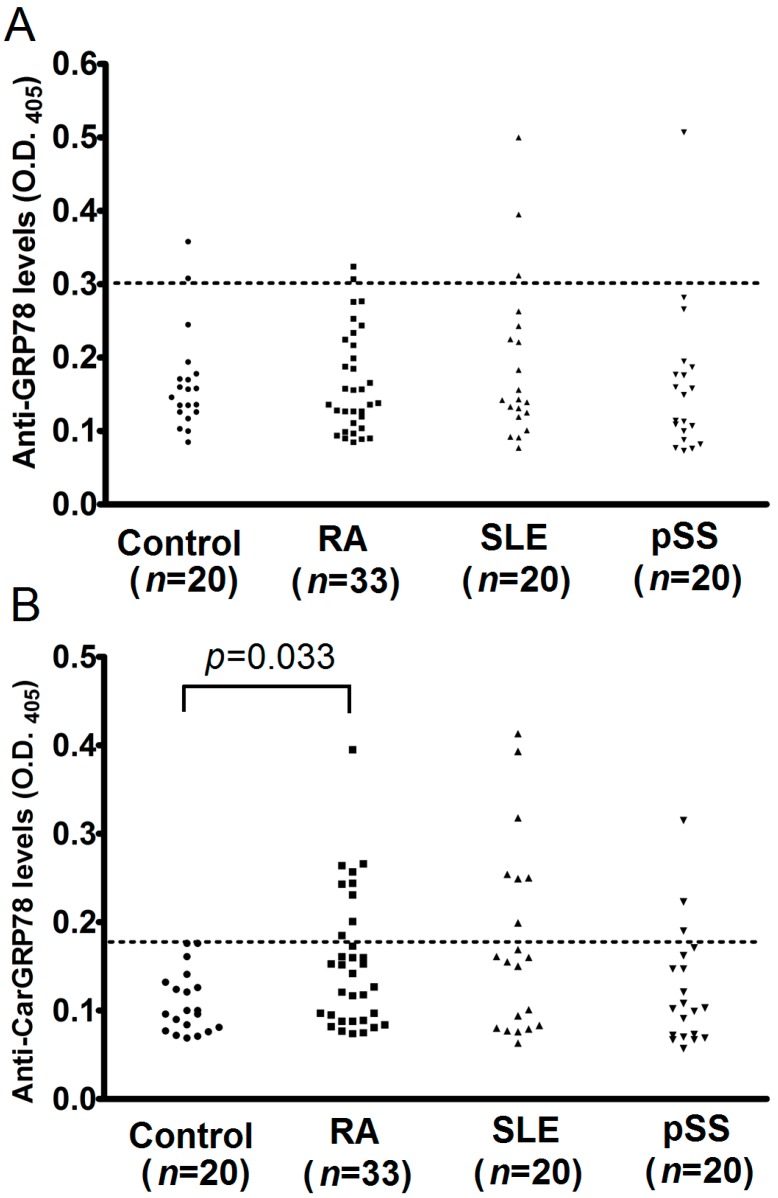
Comparison of anti-GRP78 and anti-carbamylated GRP78 (CarGRP78) antibody levels in the sera from controls, and patients with rheumatoid arthritis (RA), systemic lupus erythematosus (SLE) or primary Sjögren syndrome (pSS). (**A**) We observed no significant differences in anti-GRP78 antibody levels in the serum between patients with RA, SLE or pSS and controls; (**B**) Patients with RA but not SLE or pSS had significantly higher sera titers of anti-CarGRP78 antibodies compared with controls. O.D., optical density. The cutoff values for the anti-GRP78 and anti-CarGRP78 antibody ELISA was defined as the mean plus two standard deviations of the controls.

There were no statistically significant differences in the serum titers of anti-GRP78 antibody in patients with RA, SLE, or pSS, compared with the controls (Figure 2A). The percentage of positive of anti-GRP78 antibody was 10% (2/20) in controls, 6% (2/33) in patients with RA, 15% (3/20) in patients with SLE, and 5% (1/20) in patients with pSS.

The serum levels of anti-CarGRP78 in patients with RA was significantly higher compared with the controls (OD_405_ 0.15 ± 0.08 versus 0.11 ± 0.03, *p* = 0.033; Figure 2B). On the other hand, no significant differences were observed when comparing the serum levels of anti-CarGRP78 antibody in the controls with SLE or pSS patients (Figure 2B). The percentage of positive of anti-CarGRP78 antibody was 0% (0/20) in controls, 27% (9/33) in patients with RA, 35% (7/20) in patients with SLE, and 15% (3/20) in patients with pSS.

### 2.4. Correlation with Levels of Anti-GRP78 or Anti-CarGRP78 Antibody with Anti-CCP Antibody

**Figure 3:**
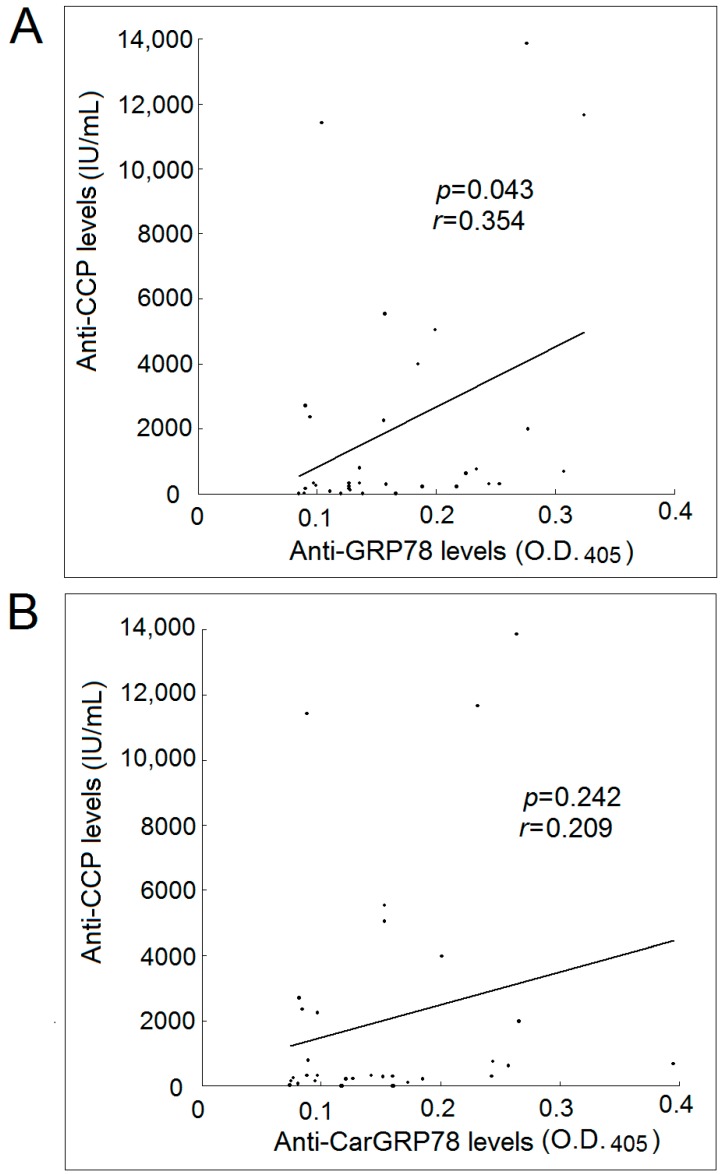
Correlation between anti-CCP levels in patients with rheumatoid arthritis and their levels of anti-GRP78 and anti-CarGRP78 antibody. (**A**) There was a statistically significant positive correlation between the anti-CCP levels and anti-GRP78 antibody levels in patients with rheumatoid arthritis; (**B**) There was no statistically significant correlation between the anti-CCP levels and anti-CarGRP78 antibody levels in patients with rheumatoid arthritis. O.D., optical density.

We found a positive correlation between the serum levels of anti-GRP78 antibody and the levels of anti-CCP antibody in patients with RA (Figure 3A). However, there were no significant correlations between the serum levels of anti-CarGRP78 antibody and the levels of anti-CCP antibody in patients with RA.

### 2.5. Correlation with Levels of Anti-GRP78 or Anti-CarGRP78 Antibody with CRP Levels

**Figure 4:**
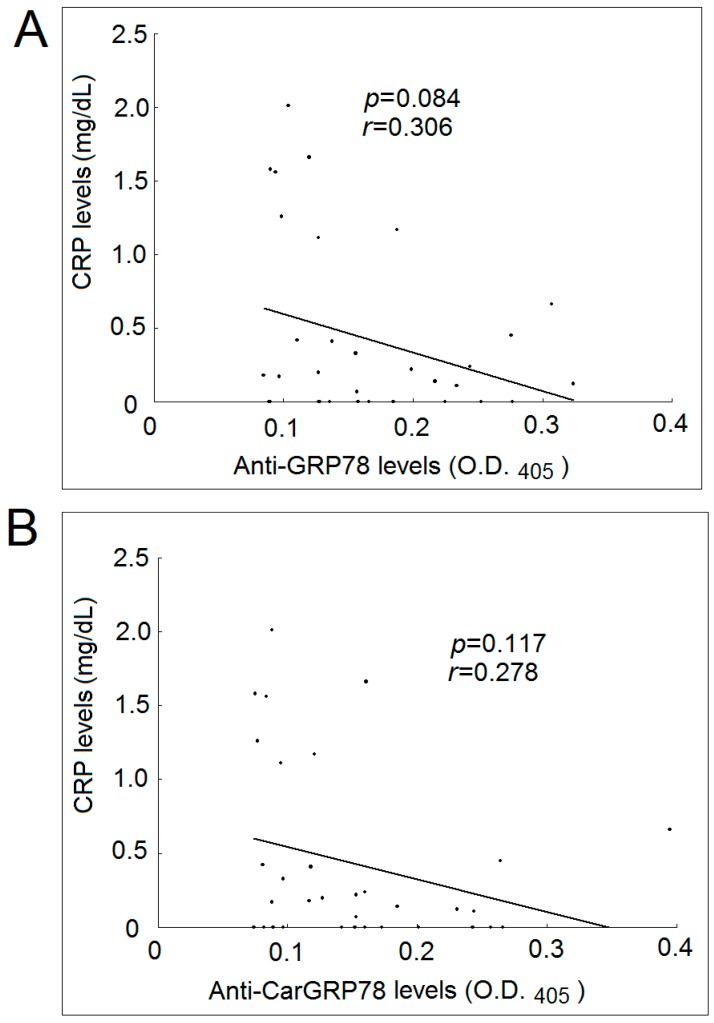
Correlation between CRP levels in patients with rheumatoid arthritis and their levels of anti-GRP78 and anti-CarGRP78 antibody. (**A**) There was no statistically significant correlation between the CRP levels and anti-GRP78 antibody levels in patients with rheumatoid arthritis; (**B**) There was no statistically significant correlation between the CRP levels and anti-CarGRP78 antibody levels in patients with rheumatoid arthritis. O.D., optical density.

We also found that there were no significant correlations between the serum level of anti-GRP78 antibody or anti-CarGRP78 and the CRP levels in patients with RA (Figure 4).

## 3. Discussion

In this study, we demonstrated the presence of anti-CarGRP78 antibody in patients with RA. We did not find a significant difference between patients with RA and controls in the serum titer of anti-GRP78 antibody as previously described [2]. We noted that the titers of anti-GRP78 were high in few controls, which led to an elevated cutoff value. This could be explained by the source of our controls, which was rheumatology outpatients rather than healthy controls. In addition, the presence of high anti-GRP78 levels could be ethnicity-related.

Compared with controls, the titer of anti-CarGRP78 antibody was significantly increased only in patients with RA (*p* = 0.033), but not in patients with SLE or in patients with pSS. We found that the percentage of positive anti-CarGRP78 antibody was 27% in patients with RA, 35% in patients with SLE, and 15% in patients with pSS. In comparison with other studies, anti-CarP antibodies were found in 45% of Japanese patients with RA [11], 11% of patients with SLE [12], and 27% of patients with pSS [13]. Furthermore, anti-CarP antibodies could be found in serum from patients with gout, osteoarthritis, spondyloarthritis, and psoriatic arthritis, though the percentage of anti-CarP antibodies positivity was below 10% among these patients [14,15]. Thus, compared with anti-CCP, anti-CarP antibodies appeared to be less specific for a differential diagnosis of RA. Further investigations are warranted to examine the presence of anti-CarGRP78 in other systemic autoimmune diseases and arthritides.

Currently, there are still doubts about the specificity of anti-CarP antibodies. Cross-reactivity between the anti-CarP antibodies and anti-CCP might exist [9,16]. Our study showed that there was a positive correlation between the titer of anti-CCP and anti-GRP78, but not anti-CarGRP78, suggesting that anti-CarGRP78 might be different from anti-CCP, as previously reported [17].

GRP78 is an important autoantigen for RA. GRP78 on its own or GRP78-specific regulatory T cells have been considered as new therapeutic strategies for treating RA [18,19]. In this study, we did not observe any significant associations between the CRP levels and anti-GRP78 or anti-CarGRP78. Nevertheless, it is difficult to rule out the effects of potential confounders such as disease duration and use of medication in our cross-sectional study. With the elevated anti-CarGRP78 antibody levels observed in patients with RA, we still speculated that carmabylated GRP78 could play a role in the immunopathogenesis of RA.

## 4. Materials and Methods

### 4.1. Materials

Acrylamide, ammonium persulfate, ampicillin, benzamidine, calcium chloride (CaCl_2_), dithiothreitol (DTT), ethylenediaminetetraacetic acid (EDTA), glycine, imidazole, isopropyl β-d-1-thiogalactopyranoside (IPTG), Luria-Bertani (LB) broth, magnesium chloride (MgCl_2_), nickel chloride, phenylmethylsulfonyl fluoride (PMSF), polyvinylidene fluoride (PVDF) membrane, potassium chloride (KCl), sodium azide (NaN_3_), sodium bicarbonate (NaHCO_3_), sodium carbonate (Na_2_CO_3_), sodium chloride (NaCl), sodium dodecyl sulfate, tetramethylethylenediamine (TEMED), tris(hydroxymethyl)aminomethane (Tris) were obtained from Sigma-Aldrich (St. Louis, MO, USA).

### 4.2. Patients

In this study, 33 patients satisfying the 1987 American College of Rheumatology (ACR) revised criteria for the classification of RA [20], 20 patients satisfying the 1982 ACR revised criteria for the classification of SLE [21] and 20 patients satisfying the American-European Consensus Group Criteria for pSS [22] were recruited. For a strict evaluation of the clinical usefulness of the autoantibodies in the differential diagnosis of RA, instead of healthy individuals from the community, we randomly recruited 20 patients from the rheumatology outpatient clinic of our hospital with no known systemic autoimmune diseases as controls.

All participants signed informed consent. The study was approved by the institutional review board of the Dalin Tzu Chi Hospital, Buddhist Tzu Chi Medical Foundation, Taiwan (No. B10403022, 27 October 2015).

### 4.3. Preparation of GRP78 and Carbamylated GRP78

Cloning of GRP78 was based on the methods described previously with modifications [23]. Full-length GRP78 cDNA was amplified by polymerase chain reaction (PCR). The resulting products were digested with *NcoI* and *EcoRI* (New England Biolabs, Ipswich, MA, USA) and then subcloned into pET32a. *E. coli* BL21 (DE3) was then transformed with recombinant pET-32a encoding GRP78. The transformed bacteria were grown in LB broth with ampicillin (0.1 g/L) and induced with 1 mM IPTG for 4 h at 37 °C. Bacteria were harvested by centrifugation, resuspended in 100 mL of 20 mM Tris-HCl buffer (pH 7.9) containing 0.5 M NaCl, 0.2 mM PMSF, 0.02% NaN_3_, 4 mM benzamidine and 0.5 mM imidazole, and lysed using a French press. GRP78 was purified from the crude lysate by sequential separation on nickel Sepharose column. The purified protein fractions were pooled, concentrated by ultrafiltration and dialyzed against polymerization buffer (5 mM Tris-HCl (pH 7.5), 2 mM CaCl_2_, 0.1 M KCl, 1 mM MgCl_2_, and 1 mM ATP). The purified GRP78 was stored at 4 °C before use. The cloned GRP78 was carbamylated by the method described by Shi et al. [6]. The CarGRP78 was extensively dialyzed against PBS and then stored.

### 4.4. Western Blotting

CarGRP78 were detected by Western blotting using a modified anti-citrulline detection kit (Upstate Biotechnology, Lake Placid, NY, USA) [24]. Briefly, 20 ng CarGRP78 and GRP78 were immobilized on PVDF membrane after 10% sodium dodecyl sulfate-polyacrylamide gel electrophoresis (SDS-PAGE), followed by electrotransfer. Then, immobilized CarGRP78 and GRP78 were modified by 2,3-butanedione monoxime and antipyrine in a strong acid solution, according to the manufacturer’s instructions. The modified citrulline residues were detected by the rabbit polyclonal anti-modified citrulline antibodies and goat anti-rabbit IgG-horseradish peroxidase (HRP) antibody conjugate.

### 4.5. Enzyme-Linked Immunosorbent Assay (ELISA) for Anti-Cyclic Citrullinated Peptides (Anti-CCP), Anti-78-kDa Glucose-Regulated Protein (Anti-GRP78) Antibody, and Anti-Carbamylated 78-kDa Glucose-Regulated Protein Anti-CarGRP78 Antibody

Anti-CCP was detected by Quanta Lite CCP3 enzyme-linked immunosorbent assay (ELISA) kits (Inova Diagnostics, San Diego, CA, USA) in accordance to the manufacturer’s recommendations. ELISA plates (BD Biosciences, San Jose, CA, USA) were coated for 2 h at 37 °C with 1 μg/mL GRP78 or CarGRP78 in coating buffer containing 35 mM NaHCO_3_ and 15 mM Na_2_CO_3_, pH 9.6. The plates were washed with phosphate-buffered saline (PBS) for four times and blocked with PBS containing 2% skim milk overnight. Then, Quanta Lite reagents (Inova Diagnostics, San Diego, CA, USA) were used for performing ELISA according to the supplier’s protocol. The plates were read at 405 nm on an Anthos Zenyth 3100 multimode fluorometer (Anthos Labtec Instruments GmbH, Salzburg, Austria). All samples were analyzed at the same time. The absorbance values of control wells were low in all cases. The cutoff values for the anti-GRP78 and anti-CarGRP78 antibody ELISA was defined as the mean plus two standard deviations of the controls.

### 4.6. Statistical Analysis

All data were represented as mean ± standard deviation. Statistical significance was assessed with non-parametric Mann-Whitney *U* test. Linear regression analysis was conducted to evaluate the correlations between levels of anti-GRP78 or anti-CarGRP78 antibody with the levels of CRP or anti-CCP antibody. Two-tailed *p* < 0.05 was considered statistically significant.

## 5. Conclusions

In conclusion, we demonstrated the presence of anti-CarGRP78 antibody in patients with RA, SLE, and pSS. The serum titer of anti-CarGRP78 antibody was significantly elevated in patients with RA compared with controls.

## Figures and Tables

**Table 1:** Demographic characteristics of controls and patients with rheumatoid arthritis, systemic lupus erythematosus, or primary Sjögren’s syndrome.

Variable	Patients with RA (*n* = 33)	Patients with SLE (*n* = 20)	Patients with pSS (*n* = 20)	Controls (*n* = 20)
Age, mean ± SD (years)	62.5 ± 12.2	38.0 ± 14.0	55.2 ± 11.2	56.7 ± 13.7
Sex (F:M)	27:6	19:1	19:1	15:5
Anti-CCP positivity, *n* (%)	29 (87.9)	1 (5.0)	2 (10.0)	0 (0)
RF positivity, *n* (%)	25 (75.8)	5 (25.0)	11 (55.0)	0 (0)

RA, rheumatoid arthritis; SLE, systemic lupus erythematosus; pSS, primary Sjögren syndrome; SD, standard deviation; anti-CCP, antibodies to cyclic citrullinated peptides; RF, rheumatoid factor.

## Abbreviations

ACPA	anti-citrullinated protein antibody
ACR	American College of Rheumatology
anti-CarGRP78	anti-carbamylated 78-kDa glucose-regulated protein
anti-GRP78	anti-78-kDa glucose-regulated protein
BiP	binding immunoglobulin protein
CarP	carbamylated proteins
CCP	cyclic citrullinated peptides
DTT	dithiothreitol
EDTA	ethylenediaminetetraacetic acid
ELISA	enzyme-linked immunosorbent assay
HRP	horseradish peroxidase
IPTG	isopropyl β-d-1-thiogalactopyranoside
LB	Luria-Bertani
OD	optical density
PBS	phosphate-buffered saline
PCR	polymerase chain reaction
PMSF	phenylmethylsulfonyl fluoride
pSS	primary Sjögren syndrome
PVDF	polyvinylidene fluoride
RA	rheumatoid arthritis
RF	rheumatoid factor
SD	standard deviation
SDS-PAGE	dodecyl sulfate-polyacrylamide gel electrophoresis
SLE	systemic lupus erythematosus
TEMED	tetramethylethylenediamine
